# Safety and Efficacy of the First Subcutaneous ICI, Envafolimab, in the Treatment of Advanced Lung Cancer Patients: A Real‐World Study

**DOI:** 10.1111/1759-7714.70101

**Published:** 2025-06-16

**Authors:** Zixuan Dou, Li Wang, Meng Rui, Yulong Yang, Yunzhi Zhou, JieLi Zhang, Qiuhong Zhao, Mengzhao Wang, Hanping Wang, Xiaotong Zhang, Xiaoxia Cui, Xiaoyan Si, Li Zhang

**Affiliations:** ^1^ School of Medicine Tsinghua Medicine, Tsinghua University Beijing China; ^2^ Department of Respiratory and Critical Care Medicine Hebei Yanda Hospital Langfang China; ^3^ Department of Respiratory and Critical Care Medicine Emergency General Hospital Beijing China; ^4^ Beijing Aerospace General Hospital Beijing China; ^5^ Department of Pulmonary and Critical Care Medicine Peking Union Medical College Hospital, Chinese Academy of Medical Sciences, Peking Union Medical College Beijing China

**Keywords:** advanced lung cancer, Envafolimab, immune checkpoint inhibitor, immunotherapy, subcutaneous injection

## Abstract

**Background:**

Envafolimab is a novel immune checkpoint inhibitor (ICI) with several advantages due to its subcutaneous administration. Phases I and II randomized controlled trials have demonstrated promising efficacy in treating colorectal and gastric cancer. However, the safety and efficacy of Envafolimab in patients with advanced lung cancer remain to be investigated.

**Methods:**

This retrospective, multicenter, open‐label, single‐arm cohort study examined real‐world medical data from patients treated at four medical centers to assess the safety and efficacy of Envafolimab in treating patients with advanced lung cancer. The primary safety outcome was Envafolimab‐related treatment‐emergent adverse events (TEAEs) and immune‐related adverse events (irAEs). The primary efficacy outcomes included overall survival (OS) and progression‐free survival (PFS). Then, the relationship between clinical parameters and prognosis was investigated using univariate and multivariate regression analyses. Furthermore, the impact of tumor EGFR driver mutation status and PD‐L1 expression on prognosis was explicitly explored in patients with nonsmall‐cell lung cancer (NSCLC).

**Results:**

The cohort comprised 58 patients with a median follow‐up time of 8.3 months (from March 1, 2022, to March 7, 2024). Envafolimab‐related TEAEs and irAEs were observed in 53.4% and 27.6% of patients, respectively. No specific clinical factors were identified as being associated with irAEs. The median OS was 8.5 months (95% confidence interval [CI] 6.2–10.8), and the median PFS was 6.1 months (95% CI 3.8–8.3). For 47 patients with NSCLC, factors including ECOG PS > 2 (HR: 2.91, *p* = 0.015), Stage IV tumor (HR: 3.43, *p* = 0.043), and nonfirst‐line Envafolimab treatment (HR: 3.27, *p* = 0.026) were associated with poor prognosis.

**Conclusion:**

Envafolimab demonstrates a tolerable safety profile and favorable efficacy. With its subcutaneous formulation, Envafolimab shows promising potential for treating advanced lung cancer.

## Graphical abstract

This multicenter retrospective study evaluated the efficacy and safety of Envafolimab in advanced lung cancer patients. Envafolimab demonstrates a tolerable safety profile and favorable efficacy. For patients with nonsmall‐cell lung cancer, the analysis identified Eastern Cooperative Oncology Group Performance Status (ECOG PS), tumor stage, and line of treatment as independent predictors of therapeutic outcomes.

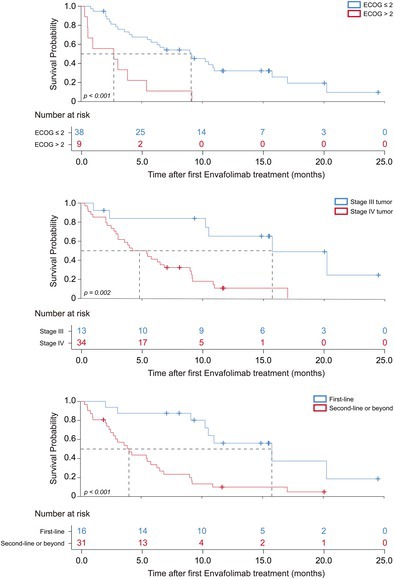

## 1 Introduction

Programmed cell death protein 1 (PD‐1) and its ligand, programmed cell death ligand 1 (PD‐L1), play crucial roles in immune checkpoint pathways that regulate T cell function. PD‐1/PD‐L1 immune checkpoint inhibitors (ICIs) disrupt the PD‐1/PD‐L1 interaction, counteracting the suppressive effects of tumor cells on T cells [1]. This intervention restores T cell cytotoxicity against tumor cells and enhances immune surveillance [2]. PD‐1/PD‐L1 ICIs including Pembrolizumab [3, 4, 5, 6, 7], Nivolumab [8], Cemiplimab [9, 10], Atezolizumab [11, 12, 13, 14], and Durvalumab [15, 16] have received FDA approval for the treatment of unresectable locally advanced or metastatic nonsmall cell lung cancer (NSCLC). In the management of extensive‐stage small cell lung cancer (ES‐SCLC), the combination of atezolizumab or durvalumab with standard chemotherapy has shown improved outcomes compared to conventional chemotherapy alone [17, 18]. These trials have confirmed the safety and efficacy of PD‐1/PD‐L1 ICIs in the treatment of advanced lung cancer.

Although the efficacy and safety of immunotherapy for patients have been extensively researched, practical issues such as time consumption, catheter‐related problems, and healthcare costs still persist. The use of subcutaneous dosage forms can significantly improve treatment convenience, reduce exposure during medical visits, and lower costs and resource utilization compared to traditional intravenous forms of immunotherapeutic drugs [19]. Multiple studies across cancer types have demonstrated patients' preference for subcutaneous administration due to reduced treatment burden [20, 21, 22]. However, only a limited number of subcutaneous antitumor agents targeting the PD‐1/PD‐L1 pathway, such as Envafolimab and Atezolizumab, are currently available [23, 24].

Envafolimab, a pioneering subcutaneous ICI, is a humanized anti‐PD‐L1 single‐domain antibody fused with a human immunoglobulin IgG1 F_c_ fragment. By binding to the PD‐L1 hotspot residues, including Ile54, Tyr56, and Arg113, through its CDR3 loop, Envafolimab exhibits a 1000‐fold higher binding capacity compared to PD‐1 [25]. Phase I clinical trials confirmed Envafolimab's favorable safety profile, with no dose‐limiting toxicity observed at the maximum dosage of 10.0 mg/kg. The drug's efficacy was indicated by an overall response rate (ORR) of 10.7%–11.6% and a disease control rate (DCR) of 34.3%–43.1% in patients with advanced solid tumors [26, 27, 28]. Pharmacokinetic studies suggest that Envafolimab can be administered every 2 weeks (Q2w) or 4 weeks (Q4w). Phase II studies have demonstrated promising results regarding the drug's safety and efficacy in patients with advanced dMMR/MSI‐H tumors, including colorectal and gastric cancers [23]. However, there is a notable absence of research on the safety and efficacy of Envafolimab for patients with advanced lung cancer.

Based on real‐world data from four medical centers in and around Beijing, this study examines the use of Envafolimab to treat locally advanced unresectable or metastatic lung cancer. Our study first provided a comprehensive analysis of Envafolimab's safety and efficacy. Then, we explored the potential clinical factors that could predict the patient's prognosis.

## 2 Methods

### 2.1 Study Design

This retrospective, multicenter, open‐label, single‐arm cohort study was designed to assess the safety and efficacy of Envafolimab in patients with locally advanced, unresectable, or metastatic lung cancer. The study reviewed the medical records of 63 patients from four medical monitoring centers between March 1st, 2022, and March 7th, 2024. Patients who provided informed consent in this study were regularly monitored for safety and efficacy following Envafolimab administration. Eligibility was determined by the following criteria: (1) age of 18 years or older; (2) a clinical or pathological diagnosis of locally advanced, unresectable, or metastatic lung cancer; (3) at least one measurable lesion based on the Response Evaluation Criteria In Solid Tumors (RECIST) version 1.1; (4) normal organ function; (5) an expected survival time exceeding 3 months. Patients with immune abnormalities were excluded (details of inclusion and exclusion criteria are listed in Supporting Information Table 1). Envafolimab was administered subcutaneously to all patients, with the dosage determined by the physician's clinical judgment. Specifically, 10 patients received a regimen of 400 mg every 3 weeks, 6 patients received 150 mg weekly, and 42 patients received 200 mg weekly. Baseline data were sourced from the most recent records at any of the monitoring centers, taken within 2 weeks before the first Envafolimab application. The follow‐up process primarily focused on safety and efficacy evaluations at the monitoring hospitals, although data from non‐monitoring hospitals were also collected via telephone follow‐up. Routine follow‐ups were scheduled bimonthly, based on patients’ visits to monitoring hospitals to document disease evaluation, adverse events, incidents, and other pertinent conditions. This study was approved by the ethics committee at Peking Union Medical Hospital. (Ethics Approval No. I‐23PJ763). This study strictly followed the Declaration of Helsinki and all applicable laws and regulations. Before their inclusion, all participants provided their informed consent through a signed consent form.

### 2.2 Descriptive Study

For safety analysis, the primary outcome was treatment‐emergent adverse events (TEAEs) and immune‐related adverse events (irAEs) related to Envafolimab. All TEAEs during the follow‐up period were documented following the guidelines of CTCAE version 5.0. Two investigators reviewed patient consultation data, primary care physician reports, and follow‐up visits to independently determine whether each TEAE was an irAE, its relevance to Envafolimab, and its severity grade. A third investigator was consulted for resolution in cases of disagreement. Each irAE was evaluated and recorded based on the guidance of *Management of immune checkpoint inhibitor‐related toxicity* from the Chinese Society of Clinical Oncology (CSCO). Specific protocols for identifying Envafolimab‐related adverse events and irAEs are detailed in Supporting Information Table 2.

For efficacy analysis, primary outcomes included overall survival (OS) and progression‐free survival (PFS). Secondary outcomes included ORR and DCR. OS was defined as the time from the patient's first dose of Envafolimab to their all‐cause death, and PFS was defined as the time from the first dose to either death or the next tumor progression. ORR and DCR were determined based on the best overall response (BOR) observed. Efficacy was assessed using RECIST v1.1 criteria by reviewing image reports from a blind independent reviewer committee. Imaging or diagnostic information from nonmonitoring hospitals was collected during routine follow‐up visits or through telephone callbacks. Patients not regularly evaluated for over 4 months or without evaluation at the end of the follow‐up time were excluded from the analysis.

### 2.3 Analytical Study

The analytical study considered predictor variables such as demographics, medical history, tumor characteristics, tumor status, prior antitumor treatments, and combination therapy with Envafolimab. Primary efficacy outcomes were defined as PFS, and safety outcomes were defined as the occurrence of irAEs. Subgroup analyses were conducted on enrolled NSCLC patients who had evaluations of the EGFR driver mutation and PD‐L1 expression before enrollment.

### 2.4 Statistical Analysis

Safety was assessed using the safety analysis set. The relationship between the occurrence of Envafolimab‐related irAEs and predictor variables was determined using univariate logistic regression. The efficacy study was conducted based on the full analysis set. OS and PFS curves were generated using the Kaplan–Meier method, and the median OS and median PFS, along with their two‐sided 95% confidence intervals (CIs), were calculated through Greenwood's formula. Differences in the OS and PFS curves were compared using the log‐rank method. Bilateral 95% CIs for ORR and DCR were calculated using the Clopper‐Pearson method. In patients with NSCLC, the relationship between the prognosis of Envafolimab treatment and predictor variables was assessed using univariate Cox regression. Only variables that were significant in the univariate analysis (*p* < 0.05) were then proceeded with the construction of multivariate models. Statistical significance was considered at *p* < 0.05 for all analyses. All statistics were performed on R (version 4.3) or SPSS (version 26).

## 3 Results

### 3.1 Patients Characteristics

**FIGURE 1:**
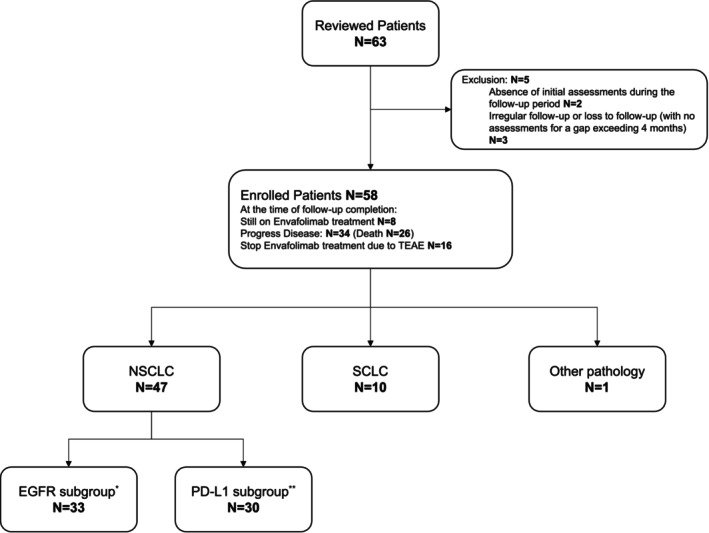
Patients enrolled in study. Patients who fulfilled the inclusion and exclusion criteria were reviewed. Five patients were excluded for indicated reasons. All enrolled patients were included in the descriptive and analytical studies. For subgroup analysis, only nonsmall cell lung cancer (NSCLC) patients were considered. *Patients diagnosed with NSCLC and assessed for the presence of EGFR driver mutations were included in the EGFR subgroup. **Patients diagnosed with NSCLC and assessed for PD‐L1 expression were included in the PD‐L1 subgroup.

A total of 63 patients diagnosed with advanced lung cancer (Stage III or IV) in four medical centers from January 2022 to March 7, 2024, were reviewed, all of whom received at least one dose of Envafolimab. Two patients were excluded due to missing initial assessments before the follow‐up cutoff, and three were excluded for irregular follow‐up, resulting in a final study population of 58 patients. The duration of follow‐up was defined as the time from the initial administration of Envafolimab to either the patient's death or the conclusion of the follow‐up period. The median follow‐up duration was 8.3 months. Of the patients, 34 experienced tumor progressions, resulting in the discontinuation of treatment, and 26 patients died during the follow‐up period. Sixteen patients halted Envafolimab due to treatment‐related adverse events, seven of which were explicitly attributed to Envafolimab toxicities (Figure 1).

A total of 47 patients were diagnosed with NSCLC, 10 patients with small cell lung cancer (SCLC), and 1 patient had an unclear pathologic diagnosis. Among the 47 patients diagnosed with NSCLC, 30 had PD‐L1 expression measured before enrollment, and 33 had EGFR targets measured (Figure 1 and Table 1). The majority of the patients enrolled were male patients (72.4%). Common past history included smoking (58.6%), hypertension (25.9%), cardiovascular disease (24.1%), and diabetes (20.7%). Most patients had Stage IV tumors (74.1%), and some had received radiotherapy before or during Envafolimab treatment (41.4%). Sixteen patients (27.6%) experienced tumor progression on other forms of PD‐1/PD‐L1 immunotherapy before enrollment. Nineteen patients (32.8%) received Envafolimab as first‐line therapy, 14 patients (24.1%) as second‐line therapy, and 25 patients (43.1%) as third‐line or beyond therapy. Chemotherapeutic agents used as coadministration in 22 patients with Envafolimab included platinum (*n* = 15), paclitaxel (*n* = 14), vinorelbine (*n* = 4), pemetrexed (*n* = 3). Patients with SCLC received etoposide (*n* = 4) or irinotecan (*n* = 3). Targeted therapy was applied in 28 cases, including anlotinib (*n* = 10), surufatinib (*n* = 7), bevacizumab (*n* = 3), and osimertinib (*n* = 4). Eleven individuals encountered pulmonary infections during Envafolimab treatment, with nine having SARS‐COV‐2 and six suspending treatments for recuperation. All six patients resumed Envafolimab treatments after recovering from infections (Table 1).

**TABLE 1:** Patients baseline characteristics.

Characteristics	*N* (%) or median (range)	
Overall	58	(100.0)
Demographic		
Gender		
Male	42	(72.4)
Female	16	(27.6)
Age (years)	67	(45–87)
Tumor pathology		
NSCLC	47	(81.0)
Adenocarcinoma	29	(50.0)
Squamous cell carcinoma	11	(19.0)
Adenosquamous carcinoma	3	(5.2)
Unclassified	4	(8.5)
SCLC	10	(17.2)
Other	1	(1.7)
Past history		
Smoking history	34	(58.6)
Hypertension	15	(25.9)
Vascular disease	14	(24.1)
Diabetes	12	(20.7)
Tumor status assessment		
ECOG		
0	0	(0.0)
1	20	(34.5)
2	28	(48.3)
3	10	(17.2)
4	0	(0.0)
Stage at Envafolimab treatment^a^		
III	15	(25.9)
IVA	20	(34.5)
IVB	23	(39.7)
Brain metastasis	16	(27.6)
Liver metastasis	8	(13.8)
Bone metastasis	20	(34.5)
Previous antitumor therapy		
Radiotherapy history	24	(41.4)
Previous PD‐1/PD‐L1 ICI therapy	16	(27.6)
Treatment lines^b^		
1	19	(32.8)
2	14	(24.1)
> 2	25	(43.1)
Coadministration with Envafolimab^c^		
Chemotherapy combined	22	(37.9)
Targeted therapies combined	28	(48.3)
Miscellaneous		
Infection during treatment^d^	11	(19.0)

*Note:* Data are presented as *n* with proportion or mean with range.

Abbreviations: ECOG, Eastern Cooperative Oncology Group; NSCLC, nonsmall cell lung cancer; SCLC, small cell lung cancer.

^a^ Staging was determined based on the 8th edition of the AJCC/UICC staging system.

^b^ Treatment lines were defined as the number of prior tumor progressions before the administration of Envafolimab plus one. Prior therapies included standard first‐line or previous‐line chemotherapy, immunotherapy, targeted therapy regimens, or combinations thereof.

^c^ Coadministration was considered when antitumor chemotherapy or targeted therapy agents were used within the time window of Envafolimab treatment.

^d^ Infection during treatment was determined if an infection was recorded within the time window of Envafolimab treatment.

### 3.2 Safety

Out of the total 58 patients, 37 (63.8%) experienced TEAE, with 31 cases (53.4%) attributed to Envafolimab. Among these, 15 patients (25.9%) encountered Grades 3–4 TEAE, of which 10 cases (17.2%) were related to Envafolimab. No incidences of Grade 5 TEAE were reported. TEAEs led to the discontinuation of Envafolimab treatment in 16 patients (27.6%), of which 12 cases (20.7%) were related to Envafolimab. The most frequently reported TEAEs were bone marrow hypocellular (19.0%), pneumonitis (13.8%), and rash (12.1%). For Envafolimab‐related TEAEs, the most common were rash (12.1%), pneumonitis (8.6%), alanine aminotransferase increased (5.2%), and anorexia (5.2%). Severe adverse events were documented in eight patients (13.8%), all related to Envafolimab, resulting in discontinuation for five patients (8.6%). Hospitalization or prolonged hospital stays were necessary for six patients (10.3%). No life‐threatening TEAE was reported (Table 2).

**TABLE 2:** Summary of adverse events during Envafolimab treatment.

	Overall		Related	
	No. of patients (%) *N* = 58			
TEAEs	37	(63.8)	31	(53.4)
Grades 3–4	15	(25.9)	10	(17.2)
Grade 5	0	(0.0)	0	(0.0)
Leads to treatment discontinuation	16	(27.6)	12	(20.7)
TEAE with incidence ≥ 10%				
Bone marrow hypocellular	11	(19.0)	2	(3.4)
Pneumonitis	8	(13.8)	5	(8.6)
Rash	7	(12.1)	7	(12.1)
SAEs	8	(13.8)	8	(13.8)
Leads to treatment discontinuation	5	(8.6)	5	(8.6)
Hospitalization (initial or prolonged)	6	(10.3)	6	(10.3)
Life‐threatening	0	(0.0)	0	(0.0)
irAEs			16	(27.6)
Grade 3			4	(6.9)
Grade 4			0	(0.0)
Leads to treatment discontinuation			9	(15.5)
Pulmonary Tox.			5	(8.6)
Dermatologic Tox.			6	(10.3)
Hepatic Tox.			2	(3.4)
Gastrointestinal Tox.			2	(3.4)
Musculoskeletal Tox.			2	(3.4)
Cardiovascular Tox.			1	(1.7)
Endocrine Tox.			1	(1.7)

*Note:* Data are presented as n with proportion. TEAEs identified during follow‐up were assessed for relevance to Envafolimab. Overall column records all TEAEs. Related column records Envafolimab‐related TEAEs.

Abbreviations: irAEs, immune‐related adverse events; SAE, severe adverse events; TEAEs, treatment emergent adverse events; Tox., toxicity.

A total of 17 patients (29.3%) experienced irAEs. Except for one case where the irAE was considered a delayed effect of prior immunotherapy, all other 16 cases (27.6%) were attributed to Envafolimab. The most frequent irAEs were dermatologic toxicity (*n* = 6), followed by pulmonary toxicity (*n* = 5), hepatic toxicity (*n* = 2), gastrointestinal toxicity (*n* = 2), and musculoskeletal toxicity (*n* = 2). Nine patients (15.5%) discontinued Envafolimab treatment due to irAEs. Four patients (6.9%) had Grade 3 irAEs, with three experiencing checkpoint inhibitor pneumonitis (CIP) and one having immune‐related hepatitis. All four patients with Grade 3 irAEs discontinued Envafolimab treatment. No Grade 4 irAE was reported (Table 2).

To investigate potential risk factors for irAE occurrence during Envafolimab treatment in advanced lung cancer patients, we analyzed demographic data, past medical history, tumor status, previous therapies, coadministered medications, and infections during treatment as variables in univariate or multivariate logistic regression analysis. The results revealed no statistical association between these variables and the occurrence of irAE (Table 3). Notably, 12 out of 16 patients (75%) who had previously experienced irAEs from prior PD‐1/PD‐L1 immunotherapy did not develop irAEs during Envafolimab treatments.

**TABLE 3:** Logistic regression: Factors associated with the occurrence of irAEs.

Characteristics	Univariate LR	
	OR (95% CI)	*p*
Demographic		
Gender	0.29 (0.06–1.44)	0.128
Age (years)	1.02 (0.96–1.08)	0.551
Past history		
Smoking history	0.87 (0.27–2.80)	0.821
Hypertension history	0.94 (0.25–3.53)	0.926
Vascular disease history	0.15 (0.02–1.25)	0.079
Diabetes history	0.85 (0.20–3.63)	0.822
Tumor pathology		
NSCLC	1.40 (0.26–7.58)	0.696
Tumor status assessment		
ECOG > 2	0.61 (0.11–3.22)	0.558
TNM Stage IV	0.74 (0.36–1.53)	0.418
Brain metastasis	0.83 (0.22–3.10)	0.786
Liver metastasis	0.86 (0.15–4.76)	0.860
Bone metastasis	0.82 (0.24–2.80)	0.749
Previous antitumor therapy		
Radiotherapy history	0.80 (0.25–2.61)	0.711
Previous PD‐1/PD‐L1 ICI therapy	1.22 (0.19–7.96)	0.833
Second‐line or beyond	1.06 (0.28–4.00)	0.926
Combined therapy with Envafolimab		
Chemotherapy combined	0.73 (0.22–2.36)	0.596
Targeted therapies combined	1.29 (0.40–4.09)	0.671
Miscellaneous		
Infection during treatment	1.07 (0.28–4.06)	0.925

*Note:* Data are presented in mean with 95% confidence interval.

Abbreviations: CI, confidence interval; ECOG, Eastern Cooperative Oncology Group performance status; LR, logistic regression; NSCLC, nonsmall‐cell lung cancer; OR, odds ratio.

### 3.3 Efficacy

**FIGURE 2:**
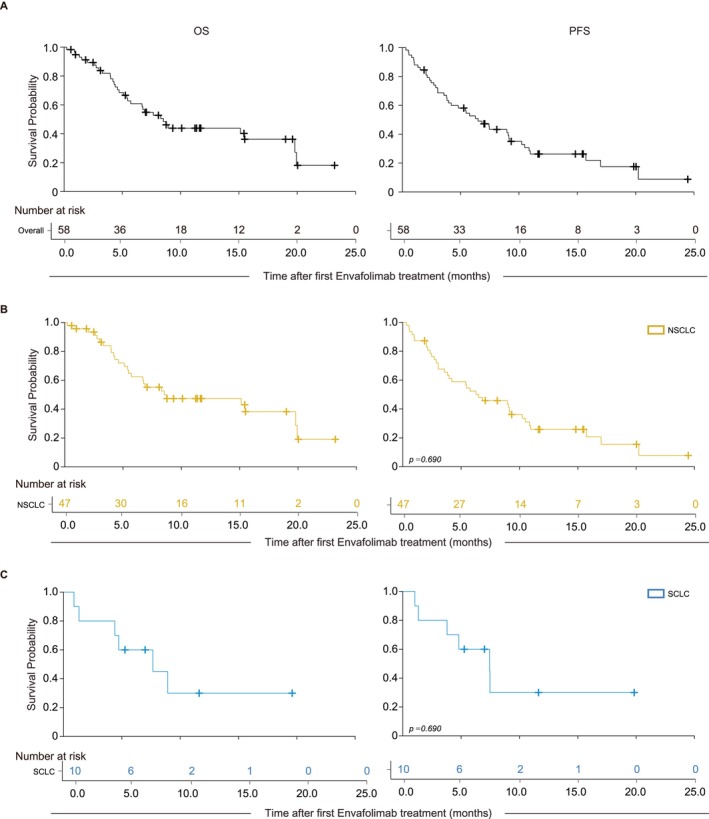
Kaplan–Meier curves of overall survival and progression‐free survival of Envafolimab treatments in advance lung cancer patients. (A) Curves of overall patients. Curves of patients in with NSCLC (B) or SCLC (C). Statistics were determined by log‐rank test. NSCLC, non‐small‐cell lung cancer; OS, overall survival; PFS, progression‐free survival; SCLC, small‐cell lung cancer.

The median OS for all patients was 8.5 months (95% CI 6.2–10.8), with a 1‐year survival rate of 43.9% (95% CI 29.8–58.0) (Figure 2 and Table 4). Patients with NSCLC had a median OS of 8.7 months (95% CI 0–19.0) and a 1‐year survival rate of 47.4% (95% CI 31.8–63.0). Patients with SCLC had a median OS of 7.7 months (95% CI 1.0–14.3) and a 1‐year survival rate of 30.0% (95% CI 0.0–63.8). The median PFS for all patients was 6.1 months (95% CI 3.8–8.3), with a 1‐year DCR of 26.5% (95% CI 14.1–39.0). Patients with NSCLC had a median PFS of 6.7 months (95% CI 4.1–9.3) and a 1‐year controlled rate of 26.0% (95% CI 12.5–39.5). Patients with SCLC had a median PFS of 6.1 months (95% CI 2.7–9.4) and a 1‐year controlled rate of 32.0% (95% CI 0.0–66.1). No significant differences in survival were observed based on pathologic types (Figure 2 and Table 4). In terms of the BOR assessment, the median DCR was 69.8% (95% CI 55.7–81.7), and the median ORR was 18.9% (95% CI 9.4–32.0) for all patients. Patients with NSCLC had a DCR of 72.1% (95% CI 56.3–84.7) and an ORR of 16.3% (95% CI 6.8–30.7). Patients with SCLC had a DCR of 66.7% (95% CI 29.9–92.5) and an ORR of 33.3% (95% CI 7.5–70.1) (Table 4).

**TABLE 4:** Summary of efficacy outcomes.

	Overall		NSCLC		SCLC	
	*N* = 58		*N* = 47		*N* = 10	
OS						
Median (months)	8.5	(6.2–10.8)	8.7	(0.0–19.0)	7.7	(1.0–14.3)
%1‐year survival	43.9	(29.8–58.0)	47.4	(31.8–63.0)	30.0	(0.0–63.8)
PFS						
Median (months)	6.1	(3.8–8.3)	6.7	(4.1–9.3)	6.1	(2.7–9.4)
%1‐year controlled	26.5	(14.1–39.0)	26.0	(12.5–39.5)	32.0	(0.0–66.1)
DCR (%)	69.8	(55.7–81.7)	72.1	(56.3–84.7)	66.7	(29.9–92.5)
ORR (%)	18.9	(9.4–32.0)	16.3	(6.8–30.7)	33.3	(7.5–70.1)

*Note:* Data are presented in median with 95% confidence interval or ratio with 95% confidence interval.

Abbreviations: DCR, disease control ratio; NSCLC, nonsmall‐cell lung cancer; ORR, objective response rate; OS, overall survival; PFS, progression‐free survival; SCLC, small‐cell lung cancer.

**FIGURE 3:**
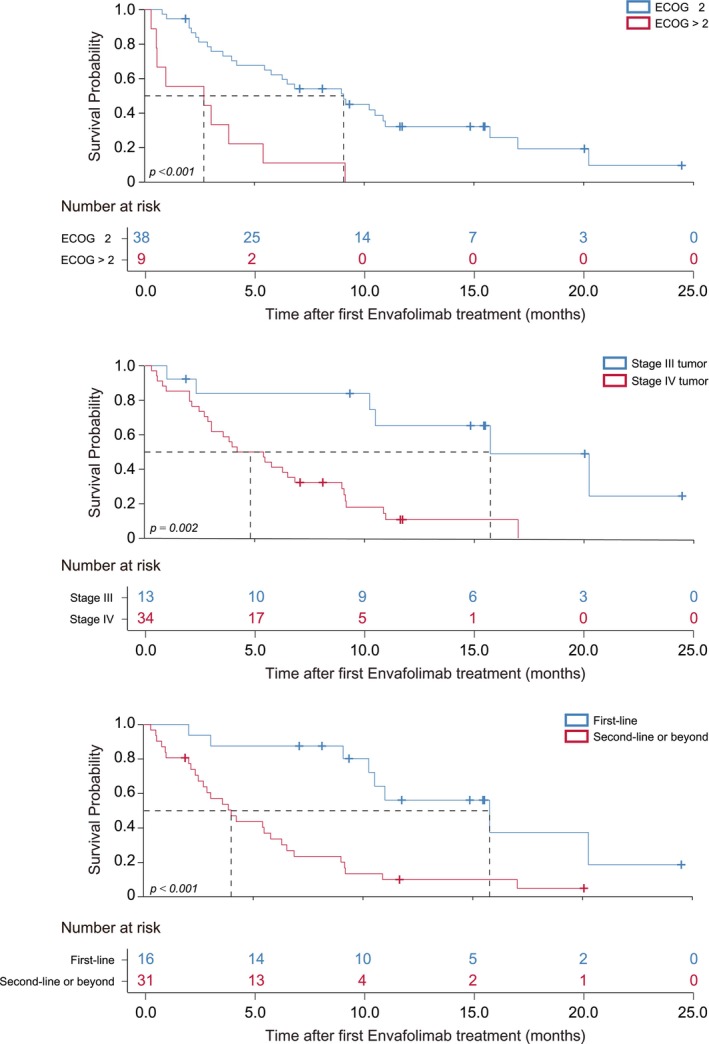
Kaplan–Meier curves of progression‐free survival according to different factors in patients with NSCLC. Statistics were determined by the log‐rank test. ECOG PS, Eastern Cooperative Oncology Group performance status.

For 47 patients diagnosed with NSCLC, risk factors associated with prognosis were investigated using a COX regression model with PFS as the outcome variable. Univariate analysis identified factors such as Eastern Cooperative Oncology Group Performance Status (ECOG PS) > 2 (hazard ratio [HR]: 4.41, *p* < 0.001), Stage IV tumor (HR: 4.89, *p* = 0.002), liver metastasis (HR: 3.19, *p* = 0.025), and nonfirst‐line Envafolimab treatment (HR: 4.49, *p* < 0.001) as correlated with poor prognosis. Multifactorial analysis confirmed that poor ECOG PS (HR: 2.91, *p* = 0.015), Stage IV tumor (HR: 3.43, *p* = 0.043), and nonfirst‐line Envafolimab treatment (HR: 3.27, *p* = 0.026) remained significantly correlated with poor prognosis (Table 5 and Figure 3). We further evaluated the prognosis of patients treated with Envafolimab as first‐line, second‐line, and third‐line or beyond therapy. In first‐line treatment, the median OS for Envafolimab was 15.5 months (95% CI 12.9–18.0), and the median PFS was 10.5 months (95% CI 9.3–11.7). When used as second‐line therapy, the median OS was 19.8 months (95% CI 0–43.5), and the median PFS was 6.5 months (95% CI 4.5–8.5). When used as third‐line and beyond therapy, the median OS was 5.1 months (95% CI 3.8–6.4), and the median PFS was 3.0 months (95% CI 1.4–4.6) (Supporting Information Table 3). The overall log‐rank test indicated a significant difference between first‐line, second‐line, and third‐line or beyond (*p* = 0.004, Supporting Information Figure 1). The Bonferroni‐corrected cohort‐by‐cohort analysis revealed a significant difference between first‐line Envafolimab treatment and third‐line or later Envafolimab treatment (HR: 3.73, *p* = 0.009). However, no significant differences were observed between first‐line and second‐line treatments (*p* = 1.000), and between second‐line and third‐line or beyond treatment (*p* = 0.117). These conclusions should be interpreted with caution due to the limitations of sample size and heterogeneity.

**TABLE 5:** Cox regression: Factors associated with poor prognosis.

Characteristics	Univariate Cox		Multivariate Cox	
	HR (95% CI)	*p*	HR (95% CI)	*p*
Demographic				
Gender	1.13 (0.54–2.37)	0.747		
Age (years)	1.00 (0.96–1.04)	0.935		
Past history				
Smoking history	0.65 (0.33–1.26)	0.198		
Hypertension history	0.87 (0.41–1.86)	0.717		
Vascular disease history	0.73 (0.35–1.53)	0.412		
Diabetes history	1.07 (0.50–2.31)	0.859		
Tumor pathology				
NSCLC	0.84 (0.28–2.48)	0.757		
Tumor status assessment				
ECOG > 2	4.41 (1.95–9.96)	0.000	2.91 (1.22–6.90)	0.015
TNM Stage IV	4.89 (1.83–13.04)	0.002	3.43 (1.04–11.37)	0.043
Brain metastasis	1.90 (0.95–3.80)	0.069		
Liver metastasis	3.19 (1.16–8.78)	0.025	2.38 (0.83–6.83)	0.107
Bone metastasis	1.75 (0.94–3.23)	0.076		
Previous antitumor therapy				
Radiotherapy history	0.80 (0.28–2.32)	0.685		
Previous PD‐1/PD‐L1 ICI therapy	1.17 (0.57–2.38)	0.667		
Nonfirst‐line treatment	4.49 (1.93–10.46)	0.000	3.27 (1.16–9.24)	0.026
Combined therapy with envafolimab				
Chemotherapy combined	0.80 (0.40–1.60)	0.535		
Targeted therapies combined	0.78 (0.40–1.53)	0.469		
Miscellaneous				
Infection during treatment	0.58 (0.26–1.30)	0.186		

*Note:* Data are presented in mean with 95% confidence intervals. In univariate analysis, factors with *p*‐value less than 0.05 were considered to have a significant difference and were then included in multifactorial analysis.

Abbreviations: CI, confidence interval; ECOG PS, Eastern Cooperative Oncology Group Performance Status; HR, hazard ratio; NSCLC, nonsmall‐cell lung cancer.

## 4 Discussion

Despite significant advances in cancer immunotherapy, the conventional intravenous administration of ICI drugs is often associated with prolonged infusion times and increased hospital exposure. Our study introduces Envafolimab as an innovative solution through its unique subcutaneous administration route. This convenient dosage form, requiring less than 1 min for each treatment session, substantially reduces costs, psychological burden, and disruptions to patients' daily routines during therapy. Particularly during the COVID‐19 pandemic, the subcutaneous administration of Envafolimab enables home‐based treatment for patients unable to visit hospitals, thereby reducing exposure risks and improving treatment accessibility. Our study highlighted the safety and efficacy of Envafolimab in patients with advanced lung cancer. Consistent with prior research, we found that Envafolimab exhibited favorable safety profiles. The Phase II RCT results showed that 84% of participants experienced Envafolimab‐related TEAEs, with 16% classified as Grades 3–5 events and 19% as SAEs [23]. Regarding irAEs, the concatenated data from three Phase I clinical studies of Envafolimab indicated an irAE incidence rate of 25.5%, with a high‐grade rate of 7.2% (Grades 3–4) [26, 27, 28]. The Phase II RCT data revealed an irAE incidence rate of 43%, with a high‐grade rate of 8% [23]. Our findings regarding the incidence rates of Grades 3–5 TEAEs, SAEs, and irAEs align with existing literature. The relatively low occurrence of overall TEAEs may be influenced by differences in cancer types, study methodologies, and patient medical histories. Notably, no new TEAEs or irAEs were identified in our study. However, pulmonary toxicity seemed to manifest at a higher frequency compared to previous RCTs (8.6% vs. 0.8%, *p* < 0.001), indicating a potential increased susceptibility of lung cancer patients to pulmonary toxicity irAEs compared to other cancer types [29, 30]. Various factors, such as concurrent medications and individual medical backgrounds, may contribute to this phenomenon. Thus, larger datasets are needed to confirm these findings for future research. Regarding efficacy assessment, no Phase II RCT studies have evaluated Envafolimab in advanced lung cancer patients. However, a comparative analysis of Phase I clinical study data showed an ORR of 11.4% in the NSCLC subgroup, which closely aligns with the outcomes reported in this paper [26, 28]. A retrospective RWS investigating Envafolimab's efficacy as part of combination therapy for various advanced solid tumors revealed an ORR of 12.0% and a DCR of 72.0% for patients with first‐line treatment, as well as an ORR of 5.1% and a DCR of 51.3% for previously treated patients [31]. Additionally, multiple RCTs exploring Envafolimab in combination with antiangiogenic agents for previously treated NSCLC patients reported ORRs ranging from 17.6% to 27.3% and DCRs from 68.4% to 84.2% [32, 33, 34]. These data presented in this study support these findings and offer a more detailed evaluation of Envafolimab's impact on lung cancer patients. In conclusion, this study highlights the safety of Envafolimab in real‐world settings for lung cancer patients, and its efficacy is consistent with previous RCTs and numerous studies, reinforcing its potential role in lung cancer therapy.

Furthermore, we revealed that a majority of patients who had experienced immunotherapy‐induced adverse events in the past did not encounter similar side effects when treated with Envafolimab. Previous research indicated a 28.8% likelihood of irAE recurrence with other immunotherapy agents, and the findings of this article support this trend in the case of Envafolimab [35]. This suggests that Envafolimab could be a viable option for immunotherapy rechallenge.

Our study provides an overall comparative evaluation of envafolimab against other immunotherapy agents that have been approved for the treatment of advanced lung cancer. Previous trials evaluating pembrolizumab reported a median overall survival (mOS) of approximately 12 months, median progression‐free survival (mPFS) of 3.7 months, and an objective response rate (ORR) of 19.4%. Specifically for first‐line treatment, pembrolizumab demonstrated a mPFS of 6.0 months with an ORR of 24.8%, accompanied by TEAEs occurring in 70.9% of patients [36]. Another study reported pembrolizumab at 10 mg/kg resulted in a mOS of 12.7 months, a mPFS of 4.0 months, and an ORR of 29% in previously treated advanced NSCLC patients, with a TEAE incidence of 66%, including Grades 3–5 events in 16% and irAEs in 19% [7]. In SCLC treatment, an RCT showed that atezolizumab combined with standard chemotherapy in first‐line advanced SCLC resulted in a mOS of 12.3 months, a mPFS of 5.2 months, and an ORR of 60.2%, with a TEAE incidence of 94.9% [17]. To reflect real‐world clinical practice more accurately, our study intentionally included patients with an ECOG PS of 2 or greater, potentially accounting for the slightly lower mOS observed compared with previous randomized controlled trial (RCT) results which typically enrolled relatively healthier patient cohorts.

More specifically, stratify analyses based on ECOG PS and treatment line provided further insights into envafolimab's clinical performance in advanced NSCLC patients. In one previous real‐world study evaluating pembrolizumab monotherapy as first‐line therapy, patients were stratified by their ECOG PS. Those with ECOG PS 0–1 had a median OS of 19.6 months (95% CI 16.0–23.1), while patients with ECOG PS ≥ 2 exhibited significantly inferior survival (mOS, 6.1 months; 95% CI 4.7–7.6) [37]. These survival outcomes were closely comparable to our own ECOG PS subgroup data (Supporting Information Table 4). The higher mOS observed in this study compared to ours might be attributed to their greater proportion of ECOG PS 0–1 patients and the use of pembrolizumab as first‐line therapy. Similarly, a real‐world study focusing on second‐line nivolumab or pembrolizumab in advanced NSCLC reported median OS and PFS of 17.9 months (537 days) and 5.0 months (150 days), respectively, with ORR and DCR being 16.93% and 60.82% [38]. Since the majority of patients in that study received immunotherapy as second‐line rather than third‐line or later treatment, their efficacy results were similar to our results with Envafolimab as second‐line therapy (Supporting Information Table 3). Furthermore, that study reported median PFS stratified by ECOG PS—patients with ECOG PS 0–1 had median PFS of 5.9 months (176 days), versus 2.3 months (70 days) for ECOG PS ≥ 2—which notably aligns with our findings [38]. These results support the non‐inferiority of envafolimab compared to pembrolizumab across different ECOG PS scores and treatment lines. Larger prospective studies are needed to precisely identify the patient populations most suitable for envafolimab treatment.

In terms of subgroup analysis on EGFR driver mutation status and PD‐L1 expression, we observed that patients with EGFR wild‐type tumors appeared to have a higher incidence of irAEs compared with those harboring EGFR driver mutations (Supporting Information Table 5). Specifically, our preliminary analysis in a small sample of 34 NSCLC patients showed that EGFR wild‐type patients were more likely to experience irAEs than EGFR driver mutation‐positive counterparts (OR: 14.44, *p* = 0.019). However, this finding might be compromised due to potential selection biases, confounding variables, and limited statistical power after examining these results via Lasso regression and IPTW. Confirmation from future larger‐scale prospective studies will be required to clearly elucidate whether EGFR mutation status truly impacts irAE risk during Envafolimab treatment. Furthermore, PD‐L1 expression data analysis suggested a trend toward longer PFS in patients with PD‐L1 expression ≥ 1% compared with PD‐L1‐negative patients (HR: 0.54; 95% CI: 0.25–1.16), though statistical significance was not reached. This reflects a potential predictive role for PD‐L1 expression levels, but larger, prospective trials are necessary to draw definitive conclusions on such associations.

This study has limitations related to the heterogeneity of patient characteristics, which primarily arises from the complex nature of real‐world cancer care. The relatively small sample size and short follow‐up period may limit the generalizability of our conclusions, particularly regarding long‐term irAEs beyond 180 days. Given these inherent limitations, careful consideration must be exercised when interpreting and comparing our findings with those of other research. Additionally, the field would benefit from prospective data collection and biospecimen analysis, particularly, with an emphasis on identifying biological markers associated with adverse events and poor prognosis.

## 5 Conclusion

This study presents for the first time the safety and efficacy of Envafolimab, the first subcutaneous ICI, in patients with advanced lung cancer. The results indicate that the outcomes of Envafolimab in real‐world applications are consistent with previous Phases I and II RCT findings. The safety and efficacy of Envafolimab in treating patients with advanced lung cancer are non‐inferior to those of currently available immunotherapies. Given the short treatment duration and the convenience of not requiring visits to medical facilities, Envafolimab offers significant advantages over traditional immunotherapies in terms of treatment convenience. These findings suggest that Envafolimab holds a promising future in lung cancer immunotherapy.

## Author Contributions

Zixuan Dou and Xiaoyan Si contributed to data analysis, statistical analysis, and manuscript preparation. Yunzhi Zhou, Jieli Zhang, Qiuhong Zhao, Li Wang, Meng Rui, Yulong Yang, Mengzhao Wang, Hanping Wang, Xiaotong Zhang, and Xiaoxia Cui contributed to clinical data acquisition. Li Zhang and Xiaoyan Si designed this study.

## Conflicts of Interest

The authors declare no conflicts of interest.

## Supporting information


**Data S1.** Supporting Information.

## Data Availability

The datasets used and analysed during the current study available from the corresponding author on reasonable request.
