# Supplementary material for: Safety and Efficacy of the First Subcutaneous ICI, Envafolimab, in the Treatment of Advanced Lung Cancer Patients: A Real‐World Study
Source: Thorac Cancer. 2025 Jun 16;16(12):e70101. doi: 10.1111/1759-7714.70101 (PMC12171066; doi:10.1111/1759-7714.70101)
Supplement: Supplementary file 1 — Data S1. Supporting Information. [file TCA-16-e70101-s001.docx]

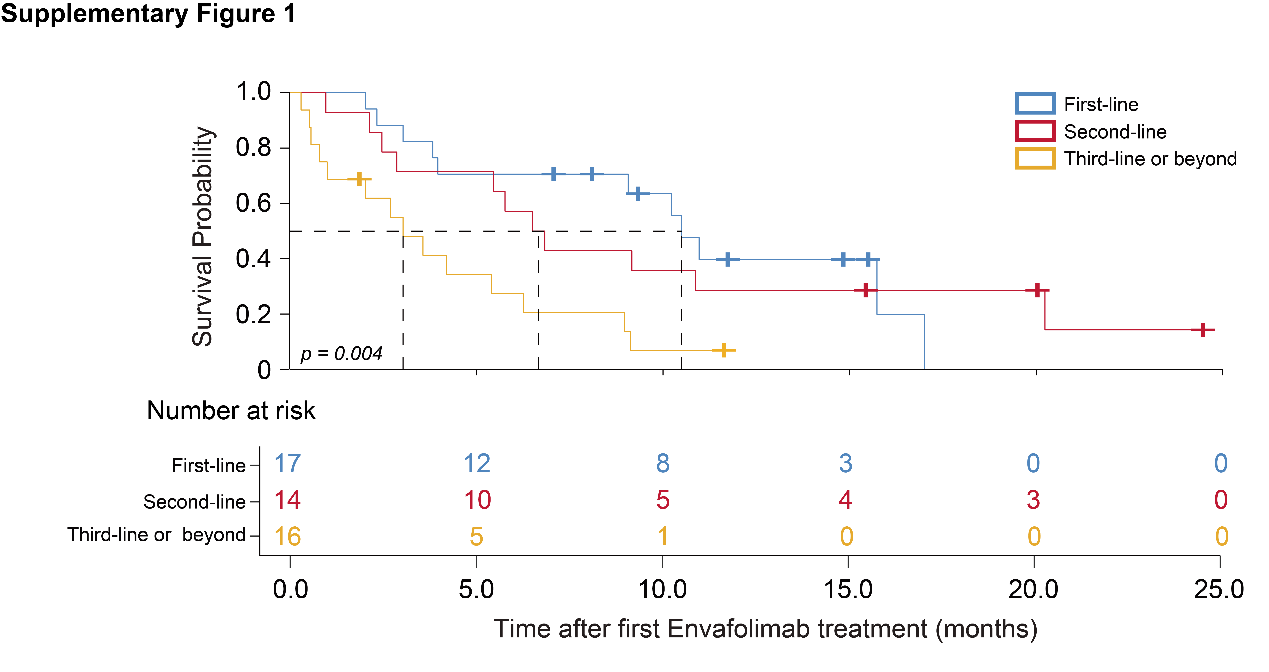


**Supplementary Figure 1 Kaplan-Meier Curves of progression-free survival according to different treatment lines in patients with NSCLC.** Statistics were determined by log-rank test.

**Supplementary Table 1 Inclusion and exclusion criteria for patients.**

| ***Inclusion Criteria*** | | |
| --- | --- | --- |
| 1 | Age ≥ 18yrs | |
| 2 | Patients are diagnosed as advanced, unresectable malignant lung tumor, confirmed by pathological tissues or cytological examination | |
| 3 | Patients have measurable lesions at baseline according to RECIST 1.1 | |
| 4 | Patients are Expected survival period over 3 months | |
| 5 | Patients have adequate organ function, confirmed by laboratory criteria | |
| 5.1 |  | ANC≥1.5×10^9/L |
| 5.2 |  | PLT≥100×10^9/L |
| 5.3 |  | TBil≤2×ULN; |
| 5.4 |  | AST and ALT≤3×ULN (for patients with liver metastasis, ALT or AST≤5×ULN is allowed) |
| 5.5 |  | SCr≤1.5×ULN; |
| 5.6 |  | Cardiac enzyme profile within the normal range |
| 6.1 | Female of childbearing age must have taken reliable contraceptive measures or undergone a pregnancy test (serum or urine) within 7 days prior to enrollment, with a negative result, and must be willing to use appropriate contraception during the trial period and for 8 weeks after the last administration of the trial medication. | |
| 6.2 | Male must agree to use appropriate contraception or have undergone surgical sterilization during the trial period and for 8 weeks after the last administration of the trial medication. | |
| ***Exclusion Criteria*** | | |
| 1 | Patients do not sign the informed consent form. | |
| 2 | Patients have known allergy or intolerance to envafolimab or its excipients. | |
| 3 | Patients were diagnosed as AIDS or other immunodeficiency disease | |
| 4 | Patients are diagnosed as active Hepatitis B (HBV DNA ≥ 500 IU/ml), Hepatitis C (positive for Hepatitis C antibodies and HCV-RNA above the lower limit of detection of the assay), or co-infection with both Hepatitis B and C. | |
| 5 | Patients has a history of allogeneic organ or bone marrow transplantation. | |
| 6 | Patients have participated in another interventional study involving administration of any immune checkpoint inhibitors or having received treatment with any immune checkpoint inhibitors within 120 days prior to the screening date. | |
| 7 | Patients’ past safety data are not accessible. | |

Abbreviation: ANC, Absolute Neutrophil Count; PLT, Platelet; TBil, Total Bilirubin; ULN, Upper Limit of Normal; AST, Aspartate Aminotransferase; ALT, Alanine Aminotransferase; SCr, Serum Creatinine; AIDS, Acquired Immunodeficiency Syndrome; HBV, Hepatitis B Virus; HCV, Hepatitis C Virus

**Supplementary Table 2 Identification criteria of Envafolimab-related TEAE and irAE**

| **Criteria of Envafolimab-related Adverse Events Identification** | | |
| --- | --- | --- |
| **Step 1** | | Adverse Event Identification Process is initiated when: |
|  | 1.1 | An unexpected event occurs during the treatment period, **AND** |
|  | 1.2 | Events are considered treatment-related, after excluding tumor progression, primary disease, and accidents |
| **Step 2** | | Events are determined to be **Envafolimab-related** if: |
|  | 2.1 | The AE was absent before Envafolimab administration, persisted during Envafolimab administration, and improved or resolved with dose reduction or discontinuation, **OR** |
|  | 2.2 | The Multidisciplinary Team consultation identifies it as an irAE. |
| **Step 3** | | Other cases are preliminarily classified as possibly Envafolimab-related, **except** when: |
|  | 3.1 | MDT consultation or clinical records explicitly state "excluded Envafolimab-induced," "excluded immunotherapy-induced," "excluded immune checkpoint inhibitor-induced," or other similar term. |
| **Step 4** | | For all events selected through Step 3, the relationship with Envafolimab was determined using following approach: |
|  | 4.1 | Two independent investigators assessed the causal relationship between AE and Envafolimab using WHO-UMC assessment criteria. In cases of discrepancy, a third expert investigator was invited to conduct an independent evaluation. Events were considered Envafolimab-related when the causality terms were classified as 'Certain,' 'Probable/likely,' or 'Possible.' |
| **Criteria of Immune-related Adverse Events Identification** | | |
| **Step 1** | | The irAE identification process is initiated when: |
|  | 1.1 | An unexpected event occurs during the treatment period, **AND** |
|  | 1.2 | Events are considered treatment-related, after excluding tumor progression, primary disease, and accidents, **AND** |
|  | 1.3 | Events are not attributed to prior treatments. |
| **Step 2** | | Events are classified as irAEs if: |
|  | 2.1 | Confirmed as irAE through MDT consultation, **OR** |
|  | 2.2 | Considered clinically related to Envafolimab **AND** either: |
|  | 2.2.1 | Response to corticosteroid and/or IVIG therapy, **OR** |
|  | 2.2.2 | Clinical suspicion of irAE, with typical manifestations as described in the CSCO immune checkpoint inhibitor management guidelines determined by two independent investigators. In cases of discrepancy, a third expert investigator was invited to conduct an independent assessment. |

Abbreviation: TEAE, Treatment-emergent adverse events; MDT, Multidisciplinary Team; irAE, immune-related adverse events; WHO-UMC, World Health Organization Uppsala Monitoring Centre

**Supplementary Table 3 Efficacy outcomes on patients with NSCLC from different treatment line.**

| **.** |  | **First-line** | | **Second-line** | | **Third-line or beyond** | |
| --- | --- | --- | --- | --- | --- | --- | --- |
|  |  | **N=17** | | **N=14** | | **N=16** | |
| ***OS*** | |  |  |  |  |  |  |
|  | Median, months | 15.5 | (12.9-18.0) | 19.8 | (0-43.5) | 5.1 | (3.8-6.4) |
|  | %1-year Survival | 67.8 | (50.2-74.2) | 47.6 | (34.4-60.8) | 0.0 | (0.0-8.3) |
| ***PFS*** | |  |  |  |  |  |  |
|  | Median, months | 10.5 | (9.3-11.7) | 6.5 | (4.5-8.5) | 3.0 | (1.4-4.6) |
|  | %1-year Controlled | 39.7 | (26.5-52.9) | 28.6 | (16.5-40.7) | 0.0 | (0.0-6.6) |
| ***DCR, %*** | | 100.0 | (80.5-100.0) | 71.4 | (41.9-91.6) | 62.5 | (35.4-84.8) |
| ***ORR, %*** | | 41.2 | (18.4-67.1) | 7.1 | (0.2-33.9) | 0.0 | (0.0-20.6) |

Data are presented in median with 95% confidence interval or ratio with 95% confidence interval. Abbreviation: PFS, Progression-Free Survival; DCR, Disease control ratio; ORR, Objective Response Rate

**Supplementary Table 4** **Stratified efficacy outcomes on prognostically significant clinical indicators**

|  |  | **N** | **OS** | | **PFS** | | **DCR** | | **ORR** | |
| --- | --- | --- | --- | --- | --- | --- | --- | --- | --- | --- |
|  |  |  | *Median, months* | | *Median, months* | | *%* | | *%* | |
| ***Treatment line*** | |  |  |  |  |  |  |  |  |  |
|  | First-line | 17 | 15.5 | (12.9-18.0) | 10.5 | (9.3-11.7) | 100.0 | (80.5-100.0) | 41.2 | (18.4-67.1) |
|  | Second-line or beyond | 30 | 6.9 | (4.3-8.5) | 5.4 | (2.1-8.7) | 46.7 | (28.3-65.7) | 0.0 | (0.0-11.6) |
| ***ECOG PS*** | |  |  |  |  |  |  |  |  |  |
|  | ECOG PS > 2 | 9 | 4.0 | (2.6-5.4) | 2.7 | (1.6-3.8) | 11.1 | (0.0-48.2) | 0.0 | (0.0-35.5) |
|  | ECOG PS ≤ 2 | 38 | 15.5 | (11.7-19.3) | 9.1 | (3.6-14.6) | 78.9 | (62.7-90.4) | 18.4 | (7.7-34.3) |
| ***TNM Staging*** | |  |  |  |  |  |  |  |  |  |
|  | TNM Stage IV | 34 | 4.0 | (3.0-5.1) | 4.8 | (2.5-9.1) | 58.8 | (40.7-75.4) | 8.8 | (0.0-23.7) |
|  | TNM Stage III | 13 | 19.9 | (19.8-NR) | 15.7 | (10.2-20.2) | 84.6 | (54.6-98.0) | 30.8 | (9.1-61.4) |

Data are presented in median with 95% confidence interval or ratio with 95% confidence interval. Abbreviation: PFS, Progression-Free Survival; DCR, Disease control ratio; ORR, Objective Response Rate; NR, Not reached.

**Supplementary Table 5 Subgroup analysis:**

| **Characteristics** | **PFS** | | **irAE** | |
| --- | --- | --- | --- | --- |
|  | HR (95% CI) | *p* | OR (95% CI) | *p* |
| EGFR WT | 0.50 (0.25 - 1.16) | 0.107 | 14.44 (1.56 - 133.58) | 0.019 |
| PD-L1 > 1% | 0.54 (0.24 - 1.23) | 0.144 | 1.38 (0.29 - 6.60) | 0.691 |

Data are presented in mean with 95% confidence intervals. For PFS and irAE, HR and OR were determined by COX regression and logistic regression, respectively. Abbreviation: PFS, progression-free survival; irAE, immune-related adverse events; HR, hazard ratio; OR, odds ratio; HR, hazard ratio; OR, odds ratio; EGFR, epidermal growth factor receptor; WT, wild type; PD-L1, Programmed cell death 1 ligand 1.
